# Toward Large-Scale Ga_2_O_3_ Membranes via Quasi-Van Der Waals Epitaxy on Epitaxial Graphene Layers

**DOI:** 10.1021/acsami.1c01042

**Published:** 2021-03-12

**Authors:** Jung-Hong Min, Kuang-Hui Li, Yong-Hyeon Kim, Jung-Wook Min, Chun Hong Kang, Kyoung-Ho Kim, Jae-Seong Lee, Kwang Jae Lee, Seong-Min Jeong, Dong-Seon Lee, Si-Young Bae, Tien Khee Ng, Boon S. Ooi

**Affiliations:** † Photonics Laboratory, Computer, Electrical and Mathematical Sciences and Engineering Division (CEMSE), King Abdullah University of Science and Technology (KAUST), Thuwal 23955-6900, Saudi Arabia; ‡ Energy and Environmental Division, Korea Institute of Ceramic Engineering and Technology, Jinju 52851, Korea; § Department of Materials Science and Engineering, Pusan National University, Busan 46241, Korea; ^∥^Division of Physical Science and Engineering, ^⊥^KAUST Catalysis Center (KCC), King Abdullah University of Science and Technology (KAUST), Thuwal 23955-6900, Saudi Arabia; # School of Electrical Engineering and Computer Science, Gwangju Institute of Science and Technology, Gwangju 61005, South Korea

**Keywords:** membranes, epitaxial graphene, van der Waals epitaxy, Ga_2_O_3_, energy release rate, solar-blind photodetectors

## Abstract

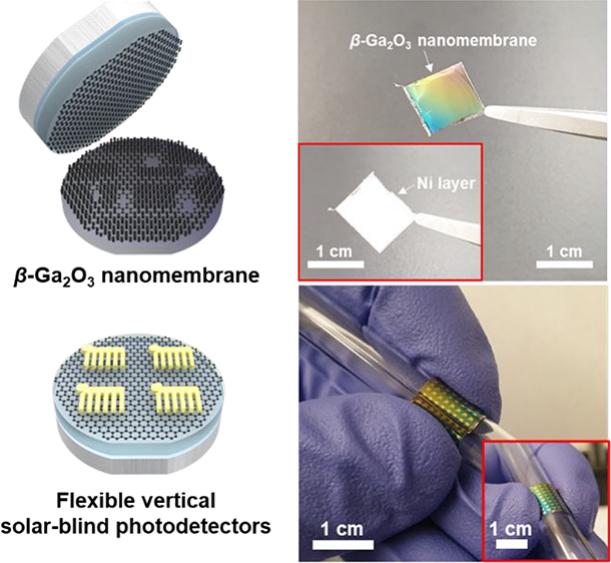

Epitaxial growth using graphene (GR), weakly bonded by van der Waals force, is a subject of interest for fabricating technologically important semiconductor membranes. Such membranes can potentially offer effective cooling and dimensional scale-down for high voltage power devices and deep ultraviolet optoelectronics at a fraction of the bulk-device cost. Here, we report on a large-area β-Ga_2_O_3_ nanomembrane spontaneous-exfoliation (1 cm × 1 cm) from layers of compressive-strained epitaxial graphene (EG) grown on SiC, and demonstrated high-responsivity flexible solar-blind photodetectors. The EG was favorably influenced by lattice arrangement of SiC, and thus enabled β-Ga_2_O_3_ direct-epitaxy on the EG. The β-Ga_2_O_3_ layer was spontaneously exfoliated at the interface of GR owing to its low interfacial toughness by controlling the energy release rate through electroplated Ni layers. The use of GR templates contributes to the seamless exfoliation of the nanomembranes, and the technique is relevant to eventual nanomembrane-based integrated device technology.

## 1 Introduction

The development of large-scale compound semiconductor membranes gives a great opportunity to make unprecedented devices such as ultralightweight, flexible, and vertical devices.^1−3^ In addition, multifunctional devices can be achieved through heterogeneous integration by transferring various kinds of membranes on one single chip.^4−6^ Despite such advantages, the successful development of membrane-based devices has been limited because it is challenging to obtain the large-scale membranes by growing three-dimensional (3D) materials on 3D materials strongly bonded by covalent bonding. Although many attempts to obtain the large-scale membranes have been performed by using laser lift-off and chemical lift-off, it also has several drawbacks as it is an extremely expensive process, and there are difficulties in finding proper sacrificial layers. To respond to the challenge, 3D materials growth by using two-dimensional (2D) materials, weakly bonded by van der Waals force, such as graphene (GR) and h-BN can be a good candidate. In other words, the considerably weaker bonding strength at the interface of 2D/2D and 3D/2D surfaces alleviates interfacial toughness and allows 3D-materials membranes peeling from the 2D materials.^7−11^ However, direct epitaxial growth of 3D materials on 2D materials, especially for GR, is not straightforward owing to its low surface energy. To overcome this hurdle, Chung et al. applied ZnO-coated layers of GR and Chen et al. used GR layers directly grown on sapphire substrates subjected to N_2_ plasma treatment for the subsequent growth of group-III-nitride materials.^12−14^ Particularly, although Chung et al. demonstrated transferable GaN-based light-emitting diodes by peeling the membranes from the GR layer, the membranes were flake-like. Nevertheless, it is clear that epitaxy using the GR layer offers a great chance to obtain large-scale membranes.

Ga_2_O_3_ has recently emerged as a promising candidate because it can be used as the absorbing layer in solar-blind photodetectors (PDs) for flame detection, and high-power electronics.^15−17^ Among the five phases of Ga_2_O_3_ (i.e., *α*, *β*, *γ*, *δ*, and ε), β-Ga_2_O_3_ has been intensively investigated owing to its thermal stability and wide band gap (∼4.9 eV) properties.^15,16^ Several studies show β-Ga_2_O_3_ grown on various substrates such as sapphire, SiC, GaN, and AlN substrates; however, these bulk substrates restrain the merits of β-Ga_2_O_3_ such as inflexibility and a difficulty to fabricate vertical devices.^18−20^

In this study, we were able to grow a 201-oriented β-Ga_2_O_3_ layer on epitaxial graphene (EG) because the EG directly interacts with SiC substrates. Based on the successful growth of the β-Ga_2_O_3_ layer on the EG, we were able to develop a large-area β-Ga_2_O_3_ nanomembrane (∼1 cm^2^) by peeling the β-Ga_2_O_3_ layer from the EG through the controlled energy release rate. We then used this nanomembrane to fabricate the flexible and vertical solar-blind PDs, which recorded a responsivity of 151.1 A/W and an improved time response (0.24 s for rise time (τ_r_) and 0.48 s for decay time (τ_d_)). The achieved performance can be attributed to the reduction of transit time of charge carriers owing to the very thin β-Ga_2_O_3_ nanomembrane that allowed vertically sandwiched electrodes on both sides. This process not only paves the way for wafer-scale exfoliation for oxide-based materials, but also provides a possibility to develop devices with unprecedented thermal, electrical, and optoelectronic properties based on the membranes.

## 2 Experimental Section

### 2.1 Preparation of Epitaxial Graphene

To obtain epitaxial graphene (EG), we prepared a 1 cm × 1 cm Si-faced 6H SiC (0001) cut from two-inch wafers. The organic residue on the substrate was removed with acetone and ethanol, and the metal residue and native oxide were removed with HCl and HF, respectively. We placed the 6H SiC substrates into a graphite box and raised the temperature to 1600 °C at a rate of 0.5 °C/s in an H_2_ atmosphere, and maintained it for 15 min to perform H_2_ etching. The pressure automatically vented to remain constant at 550 Torr, whereas it was over 550 Torr throughout the heat treatment process. After H_2_ etching, the hydrogen supply was shut-off and Ar was slowly injected. The temperature was increased to 1650 °C at a rate of 0.2 °C/s and maintained for 15 min to form the EG. The EG was finally obtained by cooling to 1000 °C while maintaining the ambient Ar, and naturally cooling to room temperature (RT).

### 2.2 Growth of β-Ga_2_O_3_ by Pulsed Laser Deposition

We attached the EG on SiC (1 cm × 1 cm) to the holder and loaded it into the load lock chamber. After placing the sample in the main chamber, the temperature was raised to 600 °C at a rate of 0.5 °C/s, and to 800 °C at a rate of 0.33 °C/s in vacuum at 10^–8^ Torr. Once the temperature had reached 800 °C, O_2_ was injected and stabilized until its partial pressure reached 5 mTorr. The distance between the Ga_2_O_3_ target and the substrate was maintained at 80 mm. The frequency of the laser pulse was set to 5 Hz and the energy per a pulse to 300 mJ. We used 20 000 laser pulses to obtain a ∼ 250 nm-thick β-Ga_2_O_3_ layer, and used 500 and 2000 laser pulses to investigate the early stage of the growth of β-Ga_2_O_3_ on both the EG and SiC. The growth of β-Ga_2_O_3_ was completed by lowering the temperature to 200 °C at a rate of 0.5 °C/s in ambient O_2_, and naturally cooling it to RT in vacuum.

### 2.3 Electroplating for Deposition of Ni Layers

Prior to the electroplating process, 50 nm-thick layers of Ti and Ni, which served as an adhesive/ohmic and a seed layer, respectively, were deposited on the β-Ga_2_O_3_ layers grown on the EG by using e-beam evaporation. The samples were attached to a homemade electroplating jig, and the electrical connection between the samples and the jig was checked with a multimeter. Particles remaining on the surface were removed by using DI rinse, and the jig with the samples was dipped into the electroplating aqueous solution (NiSO_4_). The jig connected to the sample was connected to the negative electrode, and the counter-electrode was connected to the positive electrode. The current density was controlled by increasing the voltage of the power supply. The residual stress of the Ni layer deposited by electroplating was determined by the temperature of the aqueous solution, the distance between the sample and the counter-electrode, and the current density.^21^ We used a solution at a temperature of 55 °C, an electrode distance of ∼20 cm, and a current density of 70 mA/cm^2^. The deposition rate of the Ni layer was ∼1.6 μm/min. After the deposition, the aqueous solution remaining on the sample was cleaned with the DI rinse and dried by blowing N_2_.

### 2.4 Residual Stress Measurements of Ni Layers

The Si substrates were prepared with acetone and ethanol to measure the residual stress of the electroplated Ni layers. The latter were deposited on the Si substrates using the same method of electroplating. After the deposition, two theta scans of X-ray diffraction (XRD) were first performed (Supporting Information (SI) Figure S5a). The Ni layers deposited by electroplating were polycrystalline, and the peaks of the XRD related to the Ni layers were 44.7° for (111), 52.1° for (200), 76.8° for (202), 93.5° for (311), and 99° for (222). Ni (311) was used for stress measurements because of its intense diffraction peak.^22^ The measurements of the residual stress using XRD were carried out using two theta/psi scans.^23^ In other words, we carried out two theta scans defined in the region close to Ni (311), and psi scans were performed to obtain the results of *d*-spacing versus sin^2^ψ (SI Figure S5b–d). Consequently, the residual stress of the Ni layers can be calculated by the following equation:
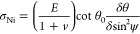
where *E*, ν, *θ*_0_, *δθ*, and ψ are Young’s modulus, Poisson’s ratio, Bragg angles with stress-free, peak shift, and the difference in angles between the normal of the specimen and that of the plane, respectively.^22^ We used 171.1 GPa as the Young’s modulus of Ni (311) and 0.3412 as its Poisson’s ratio.^22^

### 2.5 β-Ga_2_O_3_ Nanomembrane

In general, brittle materials break or spontaneously peel off when the strain exceeds a certain limit due to specific compressive or tensile stresses in the material. By contrast, stress below the limit remains internally condensed. Thus, the residual tensile stress increases the moment, and the force is transmitted downward in case of brittle substrates. In other words, when small cracks appear in part of the substrate, they advanced through mixed modes I and II fracture.^24,25^ This crack propagation can be calculated by using the energy release rate through the following formula:
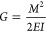
where *M*, *E*, and *I* indicate the moment, Young’s modulus, and a constant related to the Ni layer, respectively.^25^ Furthermore, when multiple layers are formed on the same substrate, there are many interfaces at each layer. They are strongly or weakly bonded with a certain interfacial toughness, and we identify the layer we can use as separation layer by calculating the interfacial toughness of each:
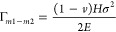
where ν, *H*, σ, and *E* indicate the Poisson’s ratio, thickness, internal residual stress, and Young’s modulus of the Ni layer, respectively.^25^ Based on the above equation, we obtained an interfacial toughness of 1.71 J/m^2^ for spontaneous exfoliation through the thickness and residual stress of the electroplated Ni layers.

### 2.6 Conventional Lateral Solar-Blind β-Ga_2_O_3_ Photodetectors

We grew ∼250 nm-thick β-Ga_2_O_3_ layers on bulk SiC substrates. In light of the β-Ga_2_O_3_ layers grown on SiC, the positive photoresist (AZ 5214) was coated at 3000 rpm for 30 s and cured at 110 °C for 1 min. We then formed mesa patterns by exposing the samples for 50 ms. The mesa structures were formed by developing the samples using a developer (AZ 726MIF) for 40 s and dry etching through inductively coupled plasma etching. We completed the mesa structures of the β-Ga_2_O_3_ layers by stripping the rest of the photoresists by acetone. For metallization, the lift-off resist (LOR) was coated at 3000 rpm for 30 s and cured at 110 °C for 5 min. Following this, we coated the LOR with AZ 5214 at 3000 rpm for 30 s and cured it at 110 °C for 1 min. The sample was exposed for 50 ms, with patterns of two laterally aligned fingers by using a direct writer and were developed for 45 s using AZ 726MIF. Once the pattern had formed, 30 nm-thick Ti and 100 nm-thick Au were deposited by e-beam evaporation, and the photoresist and LOR were removed by acetone and remover PG, respectively, to complete the fabrication of conventional lateral solar-blind PDs.

### 2.7 Flexible Vertical Solar-Blind β-Ga_2_O_3_ Photodetectors

We prepared a β-Ga_2_O_3_ nanomembrane consisting of oxidized graphene/∼250 nm-thick β-Ga_2_O_3_/50 nm-thick Ti/50 nm-thick Ni/∼40-μm-thick electroplated Ni. We were able to easily control the β-Ga_2_O_3_ nanomembrane with a conventional tweezer during the fabrication process owing to the moderately thick Ni layer. We carried out the same fabrication process as in the metallization of lateral solar-blind PDs without forming the mesa structures to complete the flexible vertical solar-blind PDs.

### 2.8 Characterizations

We used Agilent 5500 for atomic force microscopy (AFM) measurements and the free software Gwyddion to process the AFM images. All scanning electron microscopy images were acquired using the Zeiss Merlin. Powder XRD and stress measurements were examined using the Bruker D2 and D8 ADVANCE, respectively, with a Cu Kα (λ = 1.5405 Å) radiation. All materials subjected to XRD were examined using the CrystalDiffract software. For measurements of the Raman spectrum, either a 473 nm Cobolt laser or a 515 nm Ar laser was applied. Lamella for transmission electron microscopy (TEM) was prepared by a focused ion beam through FEI Helios G4. The TEM images and their fast Fourier transforms were obtained by a FEI Titan ST microscope at 300 keV. Crystal models for each material were examined by the CrystalMaker software. Raman mapping was carried out using a 473 nm Cobolt laser. We set 100 μm × 100 μm as the area of the mapping and measured each spectrum in the range from 1200 to 3000 cm^–1^. In addition, we extracted the mappings of G, 2D, and 2D/G using ranges of 1570 cm^–1^ to 1605 cm^–1^ for G, and 2695 cm^–1^ to 2775 cm^–1^ for 2D, and 2D/G, respectively. We conducted X-ray photoelectron spectroscopy measurements for the EG, β-Ga_2_O_3_ grown on EG with 500 laser pulses, and β-Ga_2_O_3_ grown on EG with 2,000 laser pulses. We used ∼283 eV for SiC, ∼ 284 eV for sp^2^ bonding, ∼284.8 eV for sp^3^ bonding, ∼286 eV for C–O–C, and ∼288.5 eV for O–C=O, as carbon-related binding energy.^26,27^ We also investigated the Ga-related binding energy by using ∼1118.7 eV for Ga_2_O_3_.^28^ The photoelectrical performance of the fabricated photodetectors was tested using a broadband 500 W mercury–xenon [Hg (Xe)] arc lamp (Newport, 66142). The emitted light passed through a monochromator (Oriel Cornerstone, CS260) equipped with a UV-based diffraction grating (Newport, 74060) before illuminating the sample. The intensity of light was controlled using neutral density (ND) filters and precalibrated using an Si-based photodetector (Newport, 818-UV). The I–V characteristics of the photodetectors were extracted using a four-terminal sensing semiconductor parameter analyzer (Agilent, 4156C).

## 3 Results and Discussion

### 3.1 Epitaxy of β-Ga_2_O_3_ Layers on Epitaxial Graphene Layers

**Figure 1:**
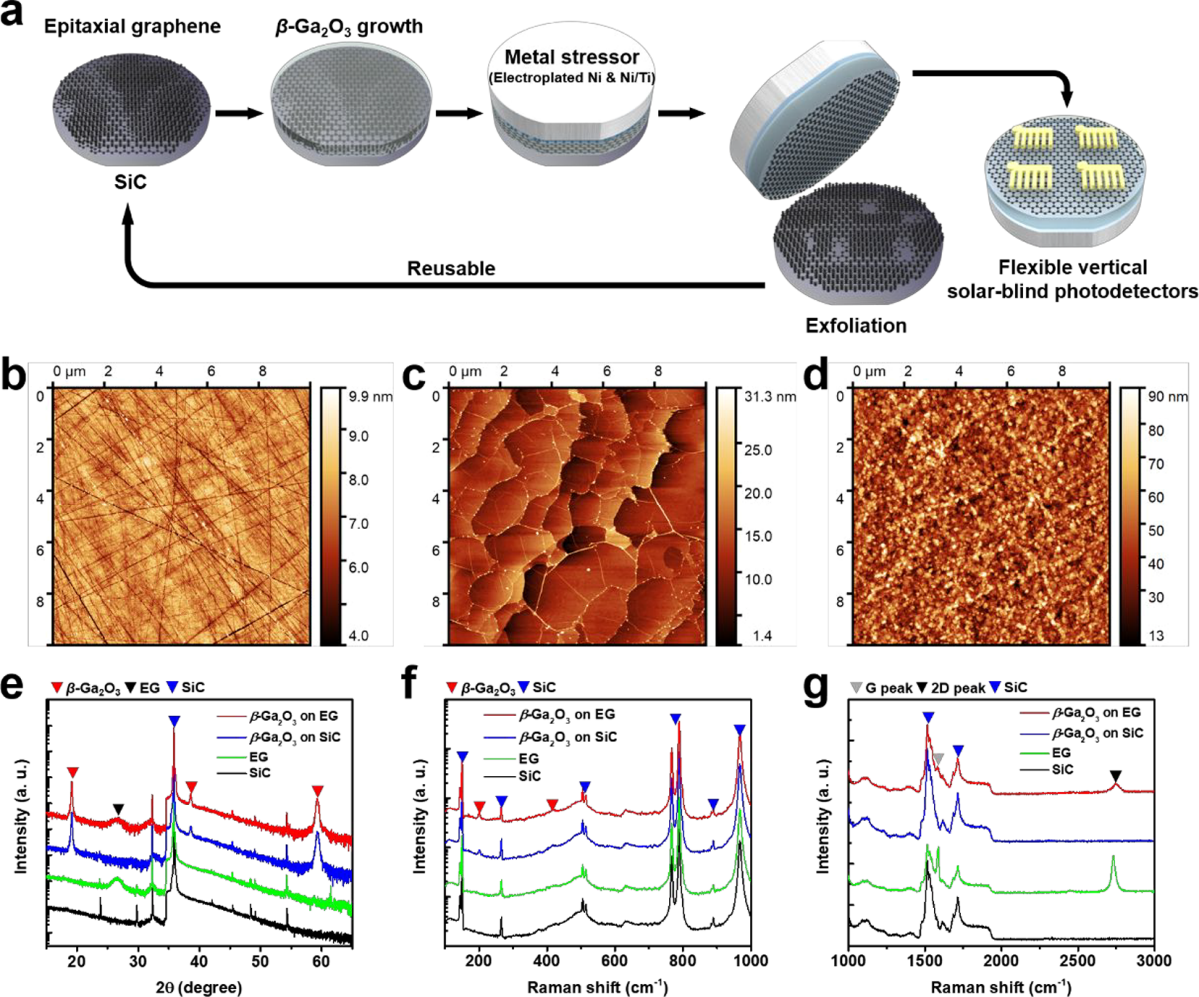
β-Ga_2_O_3_layer grown on epitaxial graphene (EG) layers. (a) Representative schematic illustration of the fabrication of the β-Ga_2_O_3_ nanomembrane and its application by using the EG layers. A β-Ga_2_O_3_ layer was grown by pulsed laser deposition using EG layers prepared by high-temperature treatment with H_2_ and Ar (i and ii). The adhesive layer (50 nm-thick Ti) and seed layer (50 nm-thick Ni) were deposited on the β-Ga_2_O_3_ by e-beam evaporation and the controlled-strained Ni layer (8–40 μm) was stacked by electroplating (iii). Because of the metal layers, the β-Ga_2_O_3_ nanomembrane was released from the EG and applied to the flexible, vertical solar-blind photodetectors (iv and v). (b), (c), and (d) show atomic force microscope images of the bare SiC substrate (SiC), EG on SiC, and β-Ga_2_O_3_ on EG, respectively. (e) Results of X-ray diffraction (XRD) ranged from 15° to 65° according to the SiC, EG, and β-Ga_2_O_3_ on SiC, and β-Ga_2_O_3_ on EG, respectively. (f and g) Results of Raman spectrum ranged from 1000 to 3000 cm^–1^, and from 100 to 1000 cm^–1^ according to the SiC, EG, and β-Ga_2_O_3_ on SiC, and β-Ga_2_O_3_ on EG.

**Figure 2:**
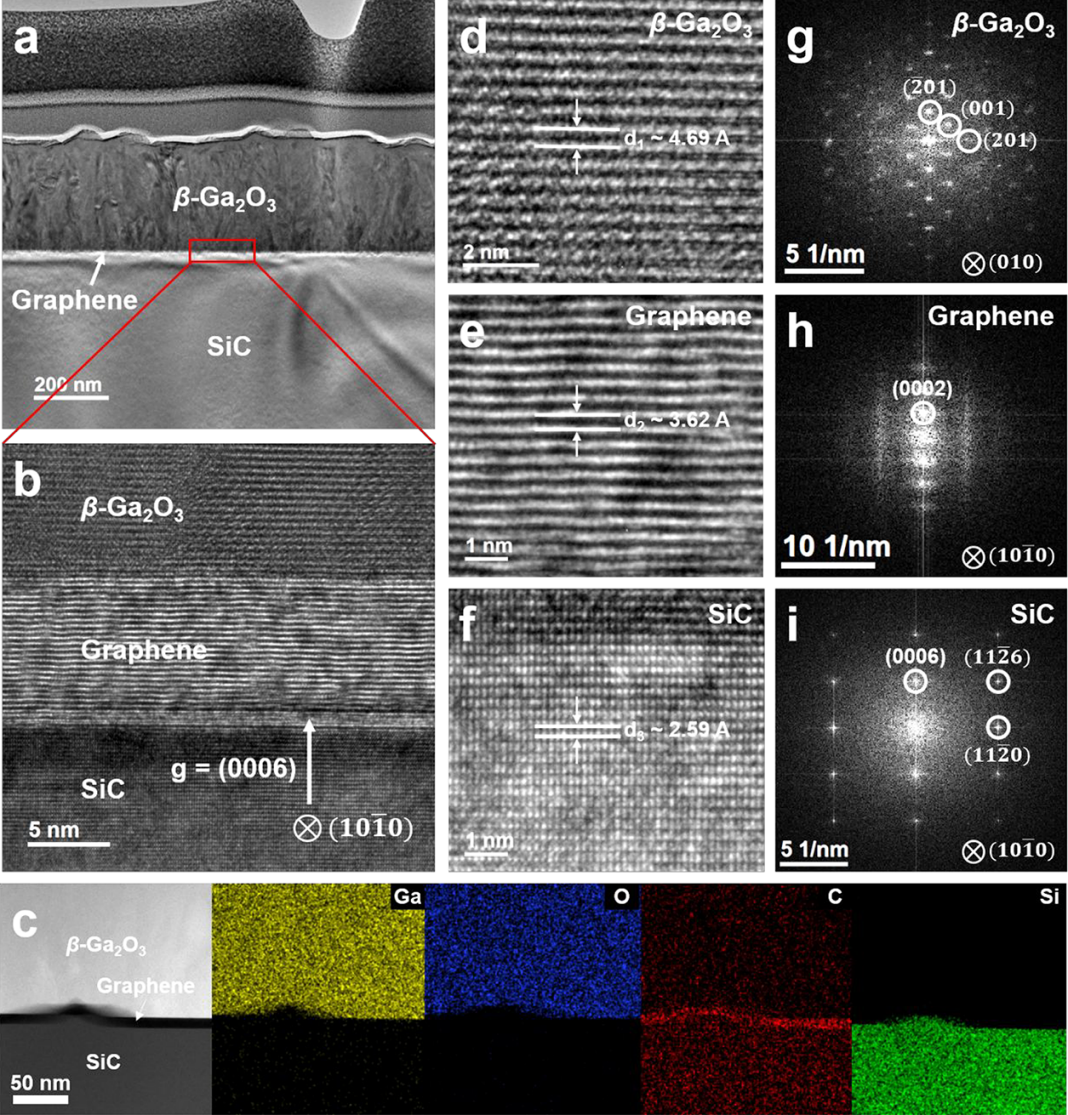
Transmission electron microscope (TEM) analysis of a β-Ga_2_O_3_layer grown on EG on SiC. (a) Low-magnification cross-sectional TEM image of carbon/Pt/carbon/Pt/Ir/β-Ga_2_O_3_/EG/SiC. The carbon/Pt/carbon/Pt/Ir layers were deposited by a sputter, an e-beam, and an ion beam to fabricate a focused ion beam (FIB) lamella. The thickness of the β-Ga_2_O_3_ was ∼250 nm. (b) High-magnification TEM image with an enlarged area for the interface of β-Ga_2_O_3_/EG/SiC. The directions of growth of graphene (GR) and β-Ga_2_O_3_ were in the (0006) plane of SiC. (c) TEM- energy dispersive X-ray images at the interface of β-Ga_2_O_3_/EG/SiC. (d–f) High-resolution TEM (HR-TEM) images of β-Ga_2_O_3_, GR, and SiC, respectively. The displacement distances of β-Ga_2_O_3_ (d_β-Ga_2_O_3__ ∼ 4.69 Å) and SiC (d_SiC_ ∼ 2.59 Å) matched well with the results in the literature (4.69 Å for β-Ga_2_O_3_ and 2.59 Å for SiC), except for GR (d_graphene_ ∼ 3.62 Å, 3.39 Å for graphite). (g–i) Fast Fourier transform images matching the HR-TEM images of β-Ga_2_O_3_, GR, and SiC.

**Figure 3:**
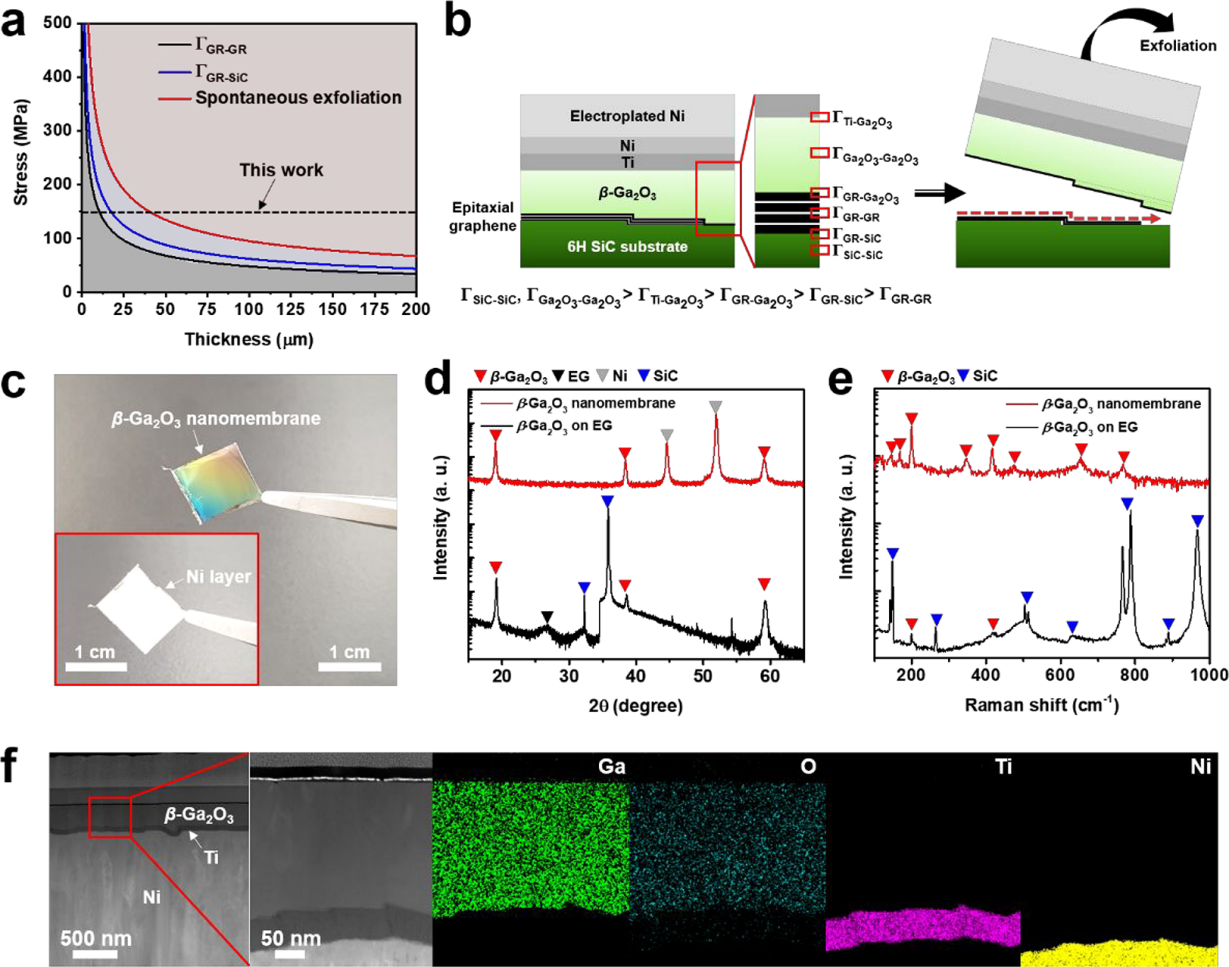
Exfoliation of a β-Ga_2_O_3_layer from EG layers. (a) Exfoliation modeling of the β-Ga_2_O_3_ nanomembrane on the EG based on the state of energy release rate. The energy release rate was related to the internal tensile stress and thickness of the Ni layers. (b) Schematic illustration explaining the principle of the exfoliation of β-Ga_2_O_3_ from the EG on SiC. There were six main interfaces from the SiC substrate to the Ti adhesive surface. The internal stress in the electroplated Ni was concentrated in EG, the smallest portion of interfacial toughness of the six interfaces, and led to the separation of the β-Ga_2_O_3_ layers from the EG. (c) Digital camera images of the exfoliated β-Ga_2_O_3_ nanomembrane; the inset indicates a flip of 180° for the nanomembrane. (d and e) Comparison of the results of XRD and Raman spectra between β-Ga_2_O_3_ grown on EG and the β-Ga_2_O_3_ nanomembrane. (f) Results of FIB and TEM-EDX for the β-Ga_2_O_3_ nanomembrane after exfoliation.

The overall process consisted of the following steps (Figure 1a): (i) formation of EG on SiC by high-temperature treatment, (ii) epitaxial growth of β-Ga_2_O_3_ layers on the EG by using pulsed laser deposition (PLD), (iii) deposition of metal layers through an e-beam evaporator for Ti and Ni, and electroplating for the Ni stressor, (iv) exfoliation of the β-Ga_2_O_3_ layers from the EG via spontaneous exfoliation, and (v) fabrication of flexible, vertical solar-blind PDs. Moreover, the EG on SiC after releasing the β-Ga_2_O_3_ layers is reusable by repetitive high-temperature treatment (not shown here).^8^ The details of each processes are included in the experimetal section. We used an atomic force microscope (AFM) to investigate the surface of a bare SiC substrate (Figure 1b). Based on AFM analysis, we then formed the EG by using a two-step high-temperature treatment.^29−31^ Following that, we observed a terrace-like morphology and grain boundaries in the EG (Figure 1c). We then grew the β-Ga_2_O_3_ layer on the bare SiC and the EG by using PLD. Although the β-Ga_2_O_3_ layer was grown by causing scratch regions to protrude in the case of the bare SiC, it was grown on over the entire area to fully cover the EG (SI Figures S1 and S2, and Figure 1d). The ∼250 nm-thick β-Ga_2_O_3_ layers on the bare SiC and EG, grown using 20 000 laser pulses, exhibited surface roughness of 10 and 12 nm, respectively (SI Figure S2c and Figure 1d). We observed no significant differences between the β-Ga_2_O_3_ layers grown on SiC and EG based on cross-sectional and surface images obtained using SEM, except for the terrace and grain boundaries on the surface of the EG (SI Figure S3). To investigate changes in the EG due to the growth of the β-Ga_2_O_3_ layer, we also prepared three samples of EG on SiC and β-Ga_2_O_3_ on EG using 500 and 2000 laser pulses and characterized by X-ray photoelectron spectroscopy (XPS) (SI Figure S1). We observed that β-Ga_2_O_3_ layers formed on the surface of the EG at the beginning of the growth, even if the graphene had been deformed. Thus, the successful growth of the β-Ga_2_O_3_ can be attributed to the adsorption of oxygen by forming oxygen plasma ambient without damaging the GR. Meanwhile, for β-Ga_2_O_3_ on the EG sample analyzed through X-ray diffraction (XRD), we obtained peaks of 19.15°, 38.6°, and 59.27° corresponding to β-Ga_2_O_3_ (201), β-Ga_2_O_3_ (402), and β-Ga_2_O_3_ (603), respectively. This indicates the growth of 201-oriented β-Ga_2_O_3_ in both cases of β-Ga_2_O_3_ on SiC and the β-Ga_2_O_3_ on the EG (Figure 1e). Thus, the β-Ga_2_O_3_ on the EG showed similar results, except GR-related peaks at 26.48°, for the β-Ga_2_O_3_ on SiC sample.^32^ In addition, we also affirmed the existence of β-Ga_2_O_3_ layers through Raman spectra (Figure 1f). However, only one peak located at 200.02 cm^–1^ for β-Ga_2_O_3_ layer due to the background of the Raman spectrum originated from the SiC substrate.^33^ However, we were able to clearly identify all peaks associated with β-Ga_2_O_3_ in the Raman spectra after the exfoliation of the β-Ga_2_O_3_ layer (Figure 3e). We observed blue shifts related to the compressive strain of our EG when initially growing the β-Ga_2_O_3_ (Figure 1g and SI Figure S1c). The blue shifts occurred even after the growth of the thin film of the β-Ga_2_O_3_ layer. Ni et al. reported that GR formed by the decomposition of SiC through heat treatment is significantly affected by the SiC substrate.^34^ Thus, GR can be formed on SiC despite the large difference in lattice constants between the former (2.47 Å) and the latter (3.07 Å) as there were matching points per 13 atoms of GR, known as the carbon mesh. Compressive strain occurred even if the carbon mesh and the SiC had matching lattice points owing to slightly different lattice constants.^34^ That is, GR epitaxially grown on SiC was strongly influenced by its lattice in the presence of compressive strain. We confirmed the ∼250 nm-thick β-Ga_2_O_3_ layer grown on the EG through low-magnification transmission electron microscopy (TEM) (Figure 2a). In addition, the high-magnification TEM and TEM-energy dispersive X-ray (EDX) clearly showed the β-Ga_2_O_3_ layer, the EG, and the SiC layer (Figure 2b,c). The high-magnification TEM image showed that the EG consisted of 20 layers of GR. Although the image showed only a small local area, we confirmed that various distribution of layers of GR were formed in our EG through Raman mapping (SI Figure S7). Three regions corresponding to the β-Ga_2_O_3_, EG, and SiC were investigated by high-resolution TEM (HR-TEM) (Figure 2d–f). The displacements of β-Ga_2_O_3_ and SiC were 4.69 and 2.59 Å, respectively, almost identical to the respective displacements of bulk β-Ga_2_O_3_ (4.68 Å) and SiC (2.54 Å). Although multilayer strain-free GR had a displacement distance of 3.39 Å, GR epitaxially grown on a SiC substrate showed a larger displacement (3.62 Å), which means that the EG exhibited compressive strain as observed in the Raman spectra. Moreover, the fast Fourier transform in various region of the HR-TEM images showed β-Ga_2_O_3_(201), EG (0002), and SiC (0006), respectively (Figures 2g–i). Through systematic examination using AFM, XRD, XPS, Raman, and TEM, we affirmed that the two main factors influencing the growth of β-Ga_2_O_3_ on the EG: (i) The Ga_2_O_3_ layers can be directly adsorbed on the surface of GR due to the effect of oxygen plasma by using PLD despite the low surface energy of GR, and (ii) unlike GR obtained by the transfer process, GR epitaxially grown on SiC acts as a buffer layer for the growth of β-Ga_2_O_3_ by replicating the lattice of the SiC substrate with compressive strain.

### 3.2 β-Ga_2_O_3_ Nanomembranes Obtained via Well-Controlled Ni Stressor

2D materials formed through the van der Waals force exhibit weak bonding compared with 3D/3D materials formed by covalent bonding. The bonding of GR is weak enough to separate its layer using only scotch tape.^35^ Although we could also have exfoliated β-Ga_2_O_3_ layers grown on EG using only the thermal release tape (TRT), we observed cracks in several areas after the exfoliation (SI Figure S4). In addition, it creates complications and difficulties in handling for scotch tape-based exfoliation method. Thus, even though exfoliation was possible using only the tape—as the interfacial toughness between GR and GR (Γ_GR-GR_) is low, 0.45 J/m^2^—there were difficulties in terms of realizing high production yield for scalable production.^36^ To overcome this drawback, we applied electroplated Ni layers with internal tensile strain to the β-Ga_2_O_3_ on the EG for obtaining an exfoliation yield of 100% by controlling the energy release rate (see Experimental Section). This method is easy to implement even in the laboratory and can increase the yield by controlling the internal stress and thickness of the Ni layers. Our structures at the interfaces of the EG showed β-Ga_2_O_3_/EG/SiC corresponding to 3D/2D/3D stacks, which could also be known as the quasi-van der Waals epitaxy. The interfacial toughness of the EG and SiC (Γ_GR-SiC_) is 0.75 J/m^2^.^37^ Comparatively, the interfacial toughness of GR and oxygen (Γ_GR-Ga_2_O_3__) is 1.47 J/m^2^.^38^ In other words, by growing the β-Ga_2_O_3_ on the EG, we were able to exfoliate β-Ga_2_O_3_ layers at the weakest interface (Γ_GR-GR_) by controlling the energy release rate via the Ni stressor (Figure 3a,b). The internal residual stress of the Ni layer used in this work was measured by the XRD stress measurement method (SI Figure S5), where this could be controlled by several factors affecting deposition through electroplating, such as temperature of the aqueous solution, current density, and the distance between the sample and the electrode (see the Experimental Section). In particular, we found a restrictive region where the energy release rate of the Ni stressor was over Γ_GR-GR_, which is when the β-Ga_2_O_3_ layers were exfoliated by using an additional supporting layer, such as TRT. In contrast, spontaneous exfoliation occurred when the energy release rate reached 1.71 J/m^2^ without any additional layer (Figure 3c and SI Figure S6). Thus, we were able to exfoliate the β-Ga_2_O_3_ layers grown from the EG based on two different methods: (i) by restricting the energy release rate between 0.45 J/m^2^ and 1.71 J/m^2^ with using TRT, and (ii) through spontaneous exfoliation by increasing the energy release rate to over 1.71 J/m^2^ (SI Figure S6). Furthermore, by directly using the EG, we avoided defects found in conventionally transferred GR, such as polymer residues, wrinkles, and voids. Through XRD measurements before and after the exfoliation, we confirmed that the β-Ga_2_O_3_ nanomembranes recorded peaks of 201-oriented β-Ga_2_O_3_ without any significant changes, except in the XRD peaks of the Ni layer (Figure 3d). Furthermore, although it was difficult to examine peaks related to β-Ga_2_O_3_ in the Raman spectra due to the background spectrum originated from the SiC substrates, we clearly observed peaks related to β-Ga_2_O_3_ after exfoliation by using the Ni stressor (Figure 3e). We can then determine that the β-Ga_2_O_3_ nanomembranes were well exfoliated, with the Ti layer used as adhesive, and Ohmic contact layers and a Ni layer used as a second substrate in cross-sectional focused ion beam (FIB)-TEM images and EDX mapping (Figure 3f). To further examine the state of the GR before/after exfoliation, we performed Raman mapping of the pristine EG on SiC, EG on SiC after exfoliation, and the back side of the β-Ga_2_O_3_ nanomembrane (SI Figure S7). The EG showed widely a distributed 2D/G ratio, which means that it was a multilayer GR (SI Figures S7a,d,g). Although most peaks related to G and 2D were similar to those of the pristine GR even after exfoliation, we did not observe any G and 2D peaks in some of the regions, and identified several empty areas in GR based on the nonexistence of 2D/G peaks (SI Figure S7b,e,h). In addition, although the GR at the back of the nanomembrane was uniformly distributed on the entire surface according to the results of 2D/G, it was oxidized based on a large change in the peak of D. This could be attributed to the fact that the GR at the top of the EG was more strongly attached to the side of β-Ga_2_O_3_ owing to the difference in the interfacial toughness (Γ_GR-Ga_2_O_3__ > Γ_GR-SiC_ > Γ_GR-GR_) at the three interfaces of β-Ga_2_O_3_/GR/GR/SiC. In addition, the surfaces of the SiC and the β-Ga_2_O_3_ after exfoliation showed a terrace, and the grain boundaries formed on the EG were equal on the side from which the β-Ga_2_O_3_ had been removed (SI Figure S8).

### 3.3 β-Ga_2_O_3_ Nanomembrane-Based Flexible Vertical Solar-Blind Photodetectors

**Figure 4:**
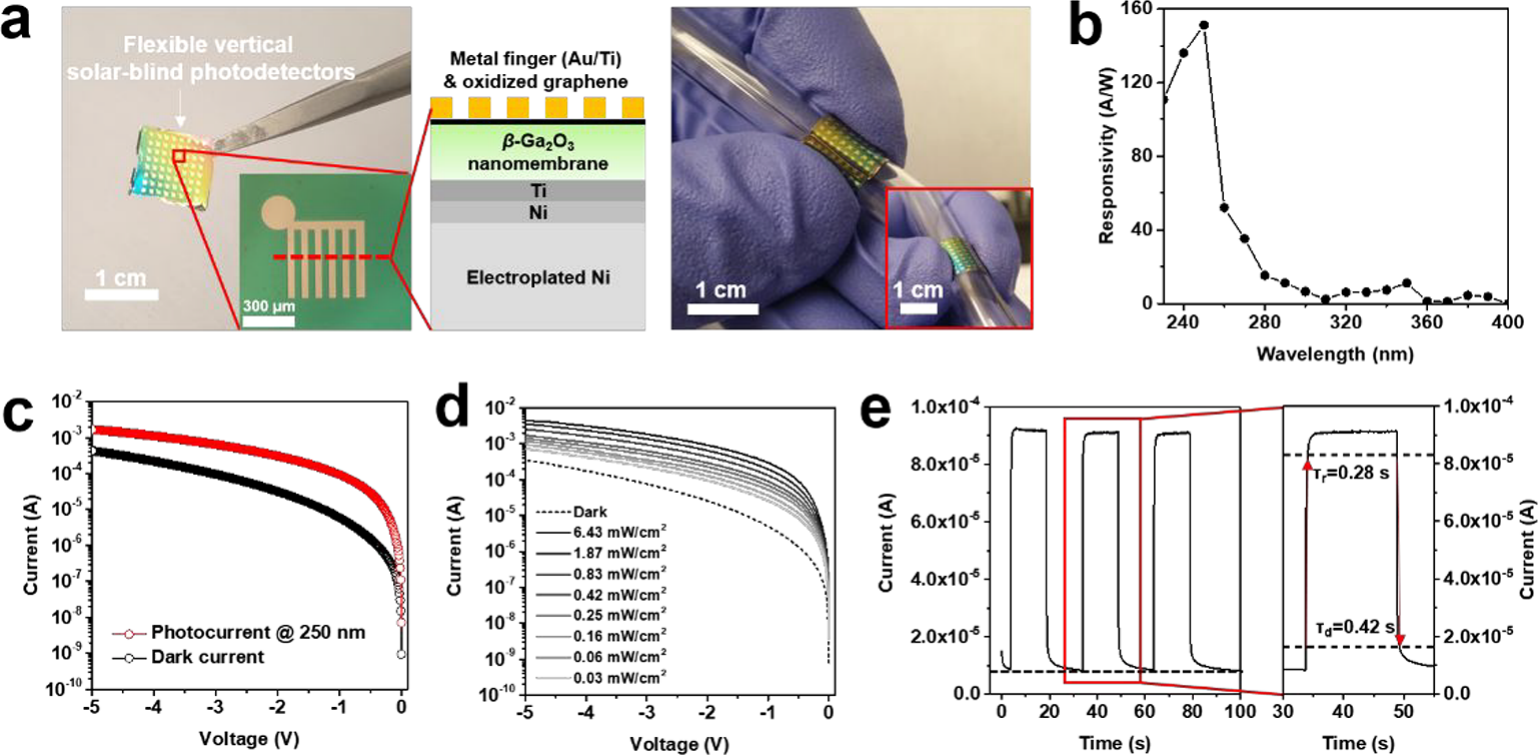
Flexible vertical solar-blind photodetectors (PDs) developed by using the β-Ga_2_O_3_nanomembrane exfoliated from the EG. (a) Digital camera images of β-Ga_2_O_3_ nanomembrane-based flexible vertical solar-blind PD arrays. The inset shows a device and its schematic illustration. The flexible vertical solar-blind PDs with a moderately thick Ni film (∼40 μm-thick Ni layer) showed outstanding flexibility, with a bending radius of 3 mm. (b) Responsivity of the flexible vertical solar-blind PDs according to wavelength. (c) Current–voltage characteristics of the flexible vertical solar-blind PDs under illumination at 250 nm (0.25 mW/cm^2^). (d) Current–voltage characteristics of the flexible vertical solar-blind PDs depending on the density of the illumination power at 250 nm. The PDs generated photocurrent even at extremely low power densities of illumination (0.03 mW/cm^2^). (e) Time-dependent photoresponse of the flexible vertical solar-blind PDs at an illumination of 250 nm.

By using the spontaneously exfoliated β-Ga_2_O_3_ with the ∼40-μm-thick Ni layer deposited by electroplating, we have also fabricated flexible, and vertical solar-blind PDs with a bending radius of ∼3 mm (Figure 4a). The electroplated Ni layer can be used as the alternative substrate with many advantages such as flexibility in comparison with bulk SiC substrate because the Ni layer is more than five times thinner and excellent electrical, optical, and thermal properties. In addition to membrane-based PDs, we have also fabricated conventional lateral-type solar-blind PDs by using a β-Ga_2_O_3_ layer grown on a bulk SiC substrate (SI Figure S9a). We measured the responsivities of the PDs at illumination wavelengths ranging from 230 to 400 nm with the interval of 10 nm (Figure 4b and SI Figure S9b). The responsivity spectrum of both the flexible vertical PDs and the lateral PDs showed photoresponses at 280 nm, with a peak responsivity identified at 250 nm, and decreased toward the 240 nm wavelength. That is, our β-Ga_2_O_3_ layers with the nanomembranes and formed on bulk SiC substrates showed the same energy band gap of ∼4.9 eV. However, the peak responsivity at 250 nm was 151.1 A/W for the membrane-based vertical PDs and 0.3 A/W for the conventional lateral PDs. Furthermore, they recorded about an order of magnitude differences in current–voltage characteristics at 250 nm (Figure 4c and SI Figure S9c). Although a weak photoresponse is recorded at low voltage that then increases with voltage in conventional lateral PDs, a large photoresponse occurred at low voltage and decreased as voltage increased in the membrane-based vertical PDs. Such results were obtained owing to the different structures of the PDs, defined along the vertical and lateral directions. Due to the distance of the metal fingers, the actual travel distance of the generated carriers in the lateral PDs is 50 μm, which could induce a higher probability of being trapped along the interdigitated metal finger, meanwhile the generated carriers in the vertical PDs is 250 nm, which is almost similar to the diffusion length of the carriers of β-Ga_2_O_3_, and thus the carriers could be efficiently extracted by forming the two metal layers as a sandwiched structure.^39,40^ We also investigated the current–voltage characteristics for varying illumination power densities at the illumination wavelength of 250 nm, and noted that the vertical PDs exhibited much higher sensitivity than the lateral PDs, based on the highest (6.43 mW/cm^2^) and lowest (0.03 mW/cm^2^) illumination power densities recorded (Figure 4d and SI Figure S9d). In general, the β-Ga_2_O_3_-based solar-blind PDs exhibited slow time-dependent photoresponse due to traps of Ga^+^ and oxygen vacancy on the surface.^41^ We also observed slow time-dependent photoresponses similar to those of current oxide-based PDs, with τ_r_ of 1.26 s and τ_d_ of 3.18 s in our lateral solar-blind PD (SI Figure S9e).^42,43^ Moreover, we observed that the charging phenomenon in which the initial dark current was not recovered occurred even though the time-dependent photoresponse was measured with a long on/off ratio of 15 s. In contrast, we obtained fast time-dependent photoresponses of 0.28 s for τ_r_ and 0.42 s for τ_d_, significantly better than those of lateral PDs in case of the vertical PDs (Figure 4e). Furthermore, the charging phenomenon was absent from the membrane-based vertical PDs. Thus, our flexible, membrane-based vertical solar-blind PDs delivered an outstanding performance in terms of responsivity and time-dependent photoresponse compared with conventional lateral PDs grown with a bulk substrate by extremely reducing the travel distance of the generated carriers via very-thin β-Ga_2_O_3_ nanomembranes.

## 4 Conclusions

Large-area epitaxial β-Ga_2_O_3_ nanomembrane (1 cm × 1 cm) was grown on EG bufferred SiC substrates, and spontaneously exfoliated from the EG, by controlling the energy release rate via an electroplated Ni layer, to realize flexible, vertical-structured solar-blind PDs. The as-fabricated PDs exhibited a higher responsivity, of 151.1 A/W, and faster time-dependent photoresponse (0.28 s for τ_r_ and 0.42 s for τ_d_) than the conventional lateral solar-blind PDs on a bulk substrate. This result can be attributed to the significant reduction in the travel distances of the generated carriers, owing to the sandwich-structured vertical PDs of thin β-Ga_2_O_3_. Our results show that EG buffer layers is a suitable template to grow oxide-based materials for producing wafer-scale oxide-based membranes through restrictive or spontaneous exfoliation by using the Ni stressor. This opens new avenues for manufacturing scalable membrane-based high-performance devices for high-power and intergrated photonic devices with unprecedented control over the opto-electrical properties.

## Supporting Information Available

XPS analysis at early stages of β-Ga_2_O_3_ on EG, AFM images for β-Ga_2_O_3_ on SiC, SEM images for surfaces and cross-sectional views, SEM images of a β-Ga_2_O_3_ nanomembrane without a Ni stressor, Results of the stress measurement, Process flowchart for the exfoliation, Raman mapping, Surface SEM images after the exfoliation, Lateral solar-blind PDs (PDF)
