# Supplementary material for: Toward Large-Scale Ga2O3 Membranes via Quasi-Van Der Waals Epitaxy on Epitaxial Graphene Layers
Source: ACS Appl Mater Interfaces. 2021 Mar 12;13(11):13410–8. doi: 10.1021/acsami.1c01042 (PMC8041250; doi:10.1021/acsami.1c01042)
Supplement: Supplementary file 1 — am1c01042_si_001.pdf [file am1c01042_si_001.pdf]

Supporting Information for

Toward Large-Scale Ga_2O_3 Membranes via Quasi-Van Der Waals Epitaxy on Epitaxial Graphene Layers

*Jung-Hong Min,[1] Kuang-Hui Li,[1] Yong-Hyeon Kim,[2] Jung-Wook Min,[1] Chun Hong Kang,[1] Kyoung-Ho Kim,[2, 3] Jae-Seong Lee,[6] Kwang Jae Lee,[4, 5] Seong-Min Jeong,[2] Dong-Seon Lee,[6] Si-Young Bae,[2] Tien Khee Ng,[1, *] and Boon S. Ooi[1, *]*

[1]Photonics Laboratory, Computer, Electrical and Mathematical Sciences and Engineering Division (CEMSE), King Abdullah University of Science and Technology (KAUST), Thuwal 23955-6900, Saudi Arabia

[2]Energy and Environmental Division, Korea Institute of Ceramic Engineering and Technology, Jinju 52851, Korea

[3]Department of Materials Science and Engineering, Pusan National University, Busan 46241, Korea

[4]Division of Physical Sciences and Engineering, [5]KAUST Catalysis Center (KCC), King Abdullah University of Science and Technology (KAUST), Thuwal 23955-6900, Saudi Arabia

[6]School of Electrical Engineering and Computer Science, Gwangju Institute of Science and Technology, Gwangju 61005, South Korea

***Corresponding Author**

Boon S. Ooi (boon.ooi@kaust.edu.sa)

Tien Khee Ng (tienkhee.ng@kaust.edu.sa)

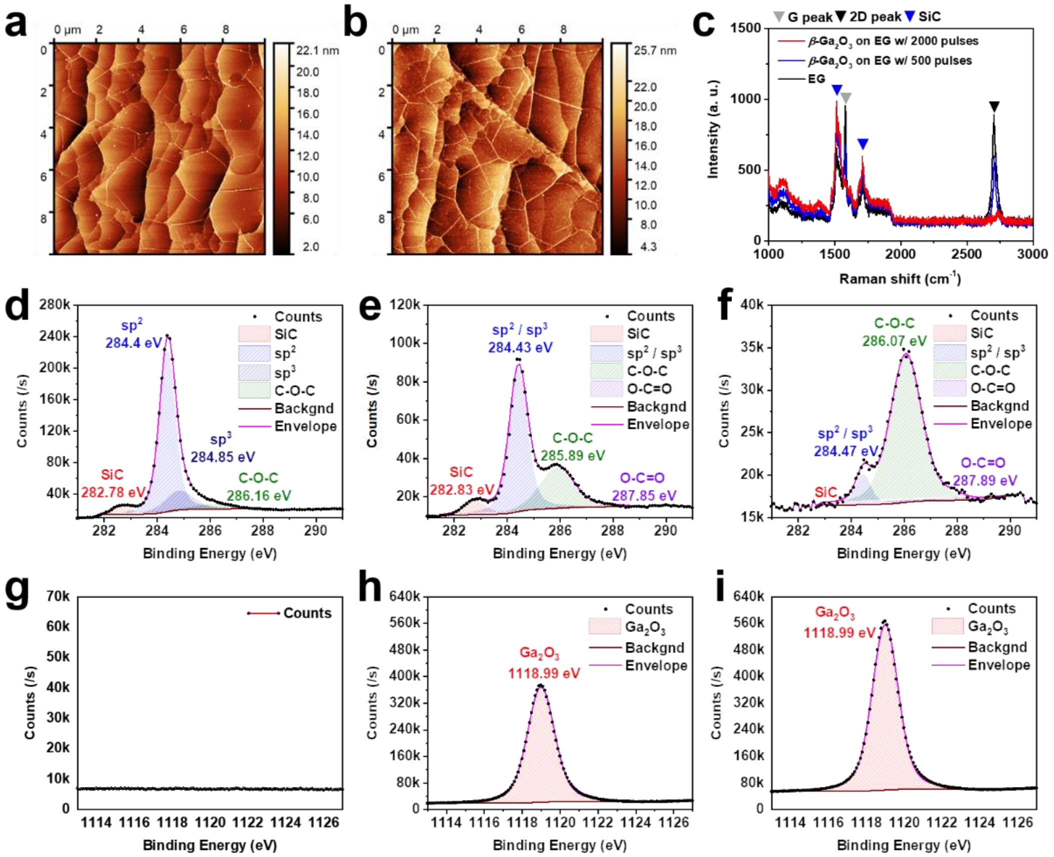

Figure S1. Investigating early stage of growth of *β*-Ga_2O_3 on epitaxial graphene layers. (a and b) Representative atomic force microscope (AFM) images of *β*-Ga_2O_3 grown on the epitaxial graphene (EG) layer with 500 and 2,000 laser pulses, respectively. The *β*-Ga_2O_3 covered the EG. (c) Results of Raman spectra related to pristine EG, (a), and (b). Although the peaks of the EG layers shifted after the growth of the *β*-Ga_2O_3, G and 2D peaks were observed, which means that the EG layers survived despite being exposed to oxygen gas at high temperature and oxygen plasma formed by Ga_2O_3 targeted by laser ablation. (d–f) Results of X-ray photoelectron microscopy (XPS) related to carbon for the EG on SiC, and *β*-Ga_2O_3 grown on the EG with 500 laser pulses (500 laser pulses) and 2,000 laser pulses (2,000 laser pulses). (g–i) Results of XPS related to Ga for EG on SiC, 500 laser pulses, and 2,000 laser pulses.

We investigated the early stage of the growth of *β*-Ga_2O_3 on the EG to identify changes in the EG by comparing the EG on SiC, and *β*-Ga_2O_3 grown on the EG with 500 laser pulses (500 laser pulses) and 2,000 laser pulses (2,000 laser pulses) through Raman spectra and X-ray photoelectron microscopy (XPS) (Figures S1c–i). Although we observed changes in the intensity, and shifts in the G and the 2D peaks subjected to 2,000 laser pulses compared with the EG on SiC subjected to 500 laser pulses, we confirmed that all samples related to the GR remained intact (Figure S1c). Moreover, we identified blue shifts in the peaks of G and 2D, which means that our EG exhibited more compressive strain than the pristine GR (1580 cm^{-1} for the peak of G and 2673 cm^{-1} for the 2D peak), even for cases of the EG (1582 cm^{-1} for the peak of G and 2702 cm^{-1} for the 2D peak) with 2,000 laser pulses (1582 cm^{-1} for the peak of G and 2742 cm^{-1} for the 2D peak).[1] To observe changes on the surface of the EG due to the *β*-Ga_2O_3 layer, we measured the EG on SiC, 500 laser

pulses, and 2,000 laser pulses through XPS. Although we observed only SiC- and sp^2 bonding-related peaks in the carbon-related binding energy for the EG on SiC, there was no peak in the Ga-related binding energy (Figures S1d and S1g). On the contrary, we were gradually able to identify oxygen bonding and sp^3 bonding in the carbon-related binding energy, and clearly observed a Ga_2O_3-related peak in the Ga-related binding energy with 500 laser pulses (Figures S1e and S1h). With 2,000 laser pulses, we observed significant changes in the peaks in the carbon-related binding energy, where the SiC-related peak disappeared and sp^3 bonding became the main peak (Figure S1f). In addition, we confirmed that a Ga_2O_3-related peak was well formed in the Ga-related binding energy (Figure S1i). That is, when β-Ga_2O_3 is grown on EG, Ga and oxygen atoms react on the surface of the EG starting at the beginning of growth.

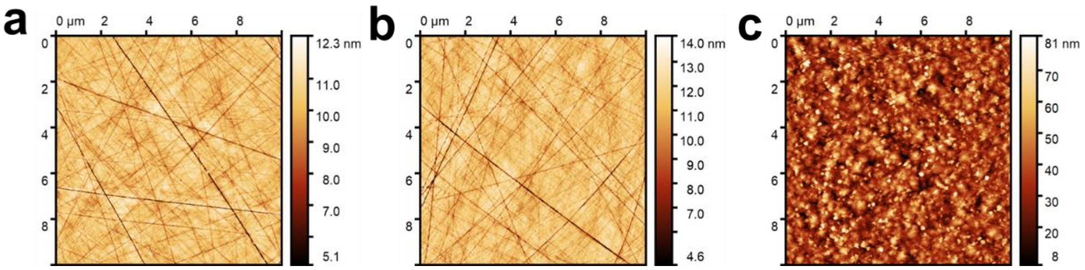

Figure S2. Changes on the surface of 6H SiC (0001) substrates following the growth of *β*-Ga_2O_3. (a and b) Representative AFM images of *β*-Ga_2O_3 grown on SiC with 500 and 2,000 laser pulses. The growth of *β*-Ga_2O_3 was carried out mainly by filling the scratch area at the beginning. (c) Representative AFM image of *β*-Ga_2O_3 grown on SiC with 20,000 laser pulses. The roughness of the *β*-Ga_2O_3 was ~10 nm.

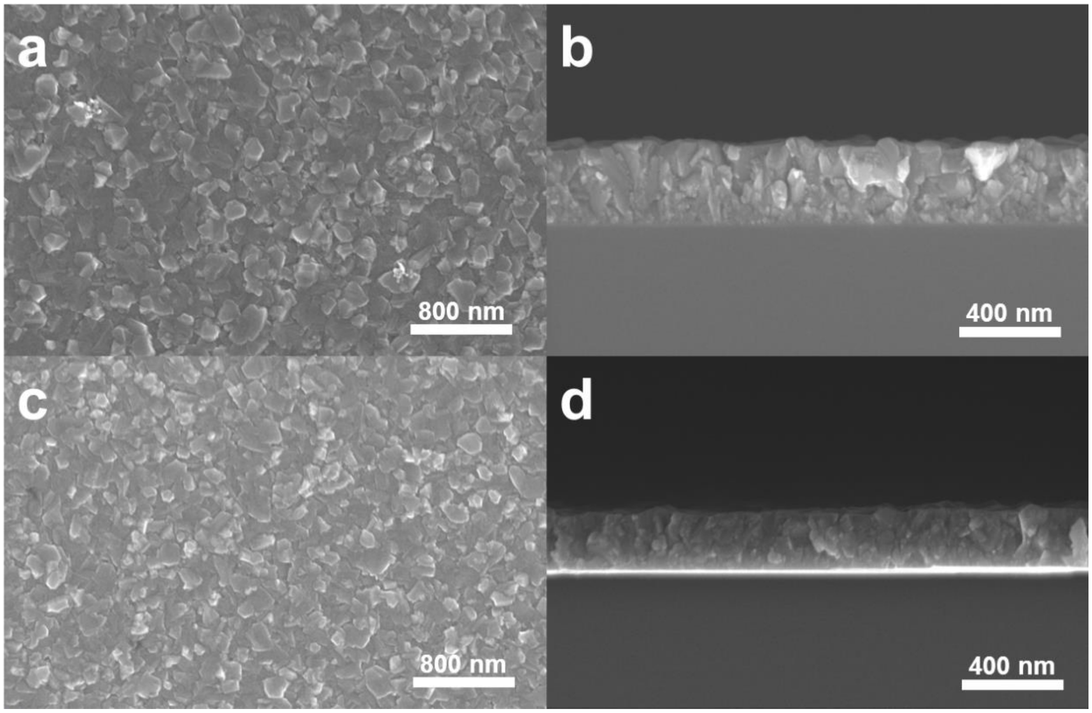

Figure S3. (a) Surface and (b) cross-sectional SEM images of β-Ga_2O_3 grown on SiC with 20,000 laser pulses. (c) Surface and (d) cross-sectional SEM image of β-Ga_2O_3 grown on the EG with 20,000 laser pulses.

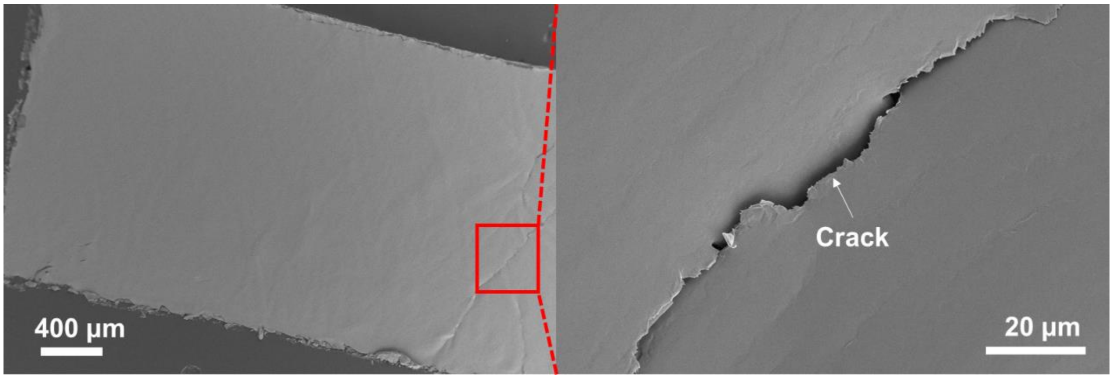

Figure S4. Surface SEM image of a *β*-Ga_2O_3 nanomembrane exfoliated by a thermal release tape (TRT), and the zoomed-in SEM image. In the case of exfoliation only by TRT, the *β*-Ga_2O_3 layers were cracked in several regions.

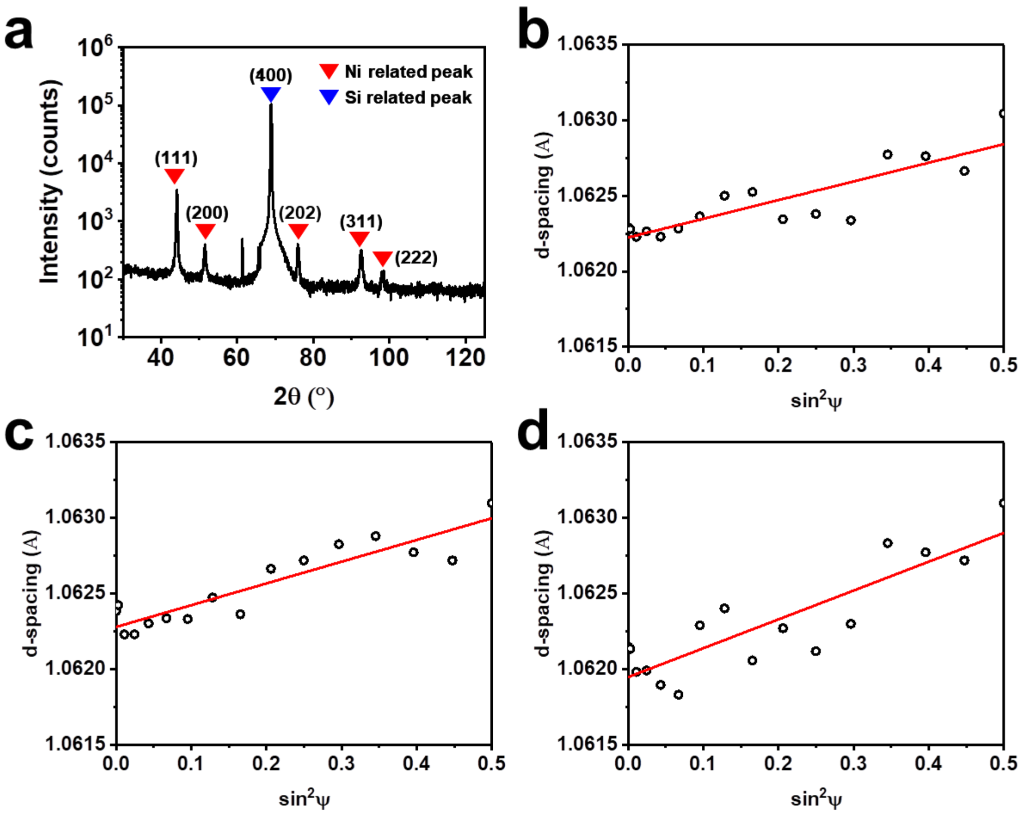

Figure S5. Stress measurement of electroplated Ni layers via X-ray diffraction. (a) 2θ scan of X-ray diffraction (XRD) ranging from 30° to 120° for an Ni layer deposited on an Si (100) substrate by using electroplating. The Ni layer was polycrystalline. (b–d) Results of d-spacing versus $\sin^2\psi$ of the reference (current density, 70 mA/cm^2; temperature of solution, 55 °C; distance between the counter electrode and the sample, 20 cm), with a decrease in distance (70 mA/cm^2, 55 °C, and 10 cm), and an increase in current density (110 mA/cm^2, 55 °C, and 20 cm), respectively. The values of d-spacing were obtained from the peak shifts of 2θ of Ni (311) by changing the degree of psi. $\sin^2\psi$ was obtained by changing the psi from 0° to 45°. The residual stress for each condition was 149 MPa for the reference, 173 MPa when the distance was reduced, and 229 MPa when current density was increased.

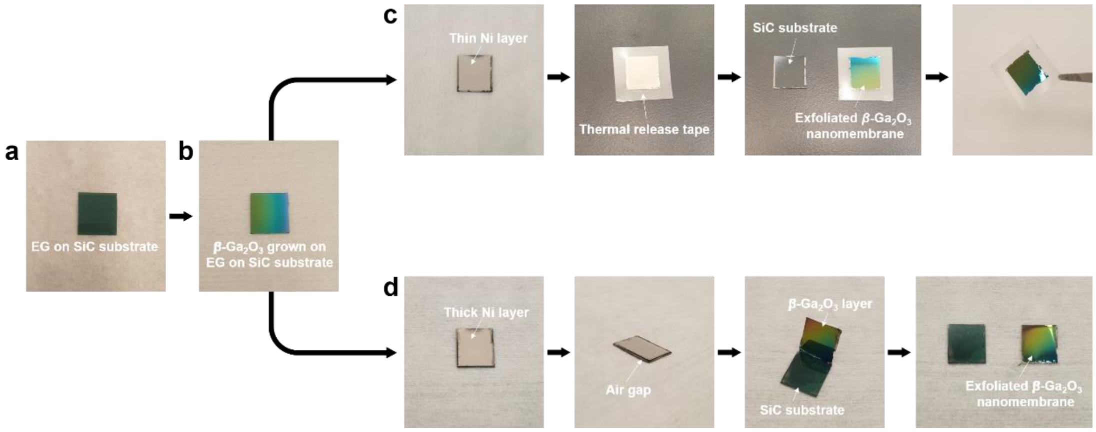

Figure S6. Process flowchart of the two methods used to obtain *β*-Ga_2O_3 nanomembrane. (a) Ready for the EG on SiC. (b) Growth of *β*-Ga_2O_3 by using the EG on SiC. (c) TRT-assisted exfoliation of *β*-Ga_2O_3 by using a thin Ni layer (~14 μm). The TRT was used as a supporting layer and handler. After depositing the thin Ni layer, the TRT was attached to the top of the Ni layer, and *β*-Ga_2O_3 layer was easily exfoliated by pulling the TRT. (d) Spontaneous exfoliation of *β*-Ga_2O_3 by using a thick Ni layer (~40 μm). After depositing the thick Ni layer, we observed an air gap between it and the SiC substrate. Thus, the *β*-Ga_2O_3 nanomembrane with the thick Ni layer was easily exfoliated by pulling the latter with tweezers. This was owing to the high energy release rate of the thick Ni layer. Furthermore, the thick Ni layer can be used as a flexible substrate for the *β*-Ga_2O_3 nanomembrane.

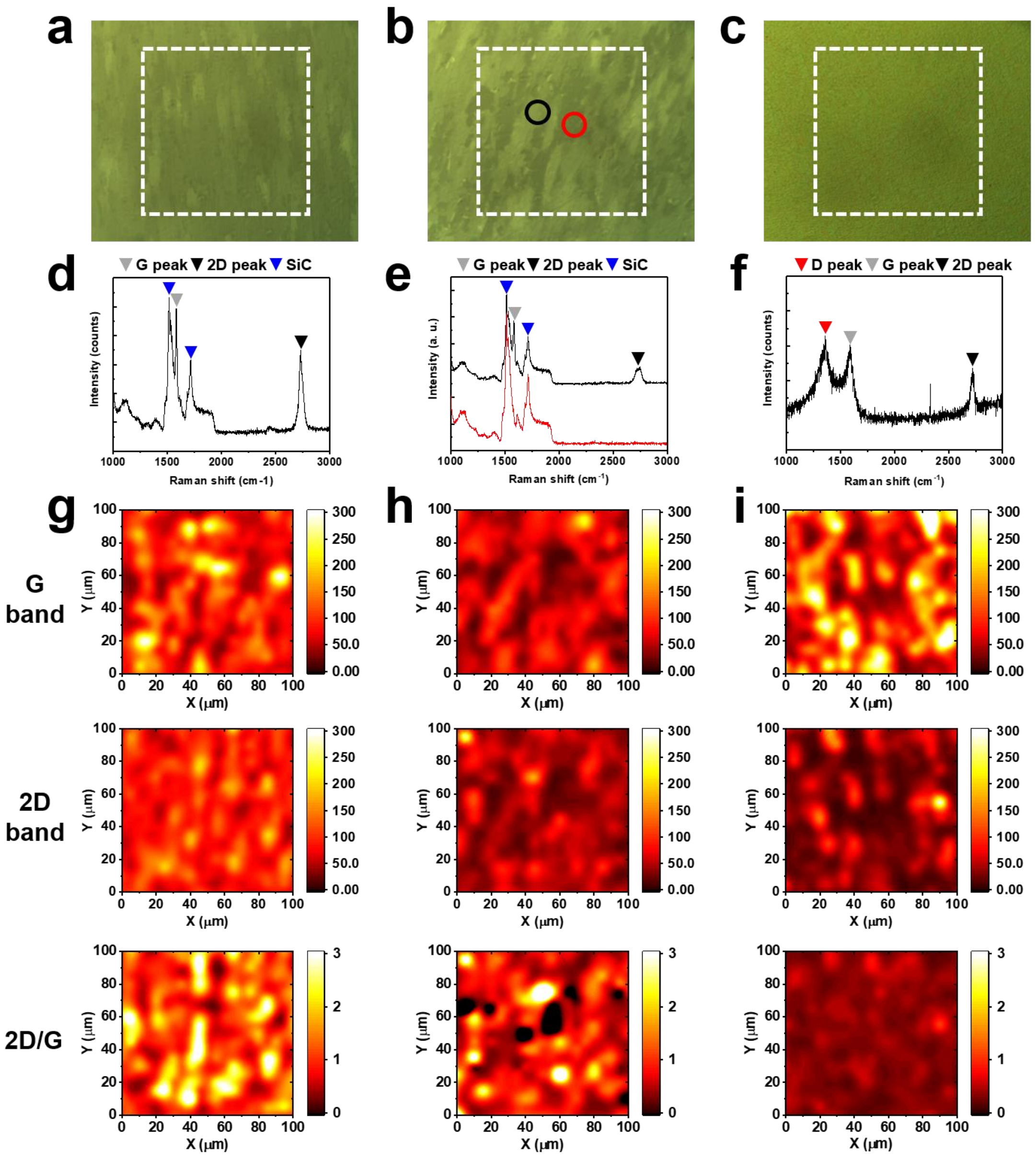

Figure S7. Graphene-related Raman analysis before and after exfoliation. (a–c) Optical microscope images of the EG on SiC, EG on SiC after exfoliation, and the back side of the β-Ga_2O_3 nanomembrane, respectively. (d–f) Representative Raman spectra of the EG on SiC, EG on SiC after exfoliation, and the back side of the β-Ga_2O_3 nanomembrane at a range of 1000~3000 cm^{-1}. In the EG on SiC sample after separation, a region with or without graphene was formed, and after separation, a single layer of graphene remained on the back side of the β-Ga_2O_3 nanomembrane and was observed to oxidize. (g–i) Results of Raman mappings of the EG on SiC, EG on SiC after exfoliation, and the back side of the β-Ga_2O_3 nanomembrane represented by white dashed areas in a–c, ranging from 1570 cm^{-1} to 1605 cm^{-1} for the G band, 2695 cm^{-1} to 2775 cm^{-1} for the 2D band, and the ratio of 2D/G, respectively.

We investigated the interfacial states related to GR before and after the exfoliation of the β-Ga_2O_3 layer through Raman mapping and SEM images (Figures S7 and S8). Although the EG formed by the heat treatment of SiC had clear G peaks (~1585 cm^{-1}) and 2D peaks (~2720 cm^{-1}) (Figure S7d), the EG used here formed in diversely distributed layers as shown by the Raman mapping of a selected optical microscope image, and represented by the white dot (Figure S7a). This is because the ratio of the 2D and G peaks (2D/G) showed varying distributions from one to three (Figure S7g). Compared with the original EG, we observed significant changes in the state of the EG on SiC after exfoliation and the β-Ga_2O_3 nanomembrane (Figures S7e and S7f). In particular, we observed an oxidized GR layer uniformly exfoliated by being attached to the β-Ga_2O_3 layer, as the D peak increased significantly and the ratio 2D/G was uniform across the entire surface (Figures S7f and S7i). This result is related to the combination of oxygen and GR, which we identified by using XPS immediately following the growth of the β-Ga_2O_3. That is, the top of the EG layer was oxidized by growing the β-Ga_2O_3 layer, and the oxidized GR layer could be detached using the Ni stressor because the value of $\Gamma_{GR\text{-}Ga_2O_3}$ was higher than those of $\Gamma_{GR\text{-}SiC}$ and $\Gamma_{GR\text{-}GR}$. On the contrary, we obtained similar G and 2D peaks to those of the original EG without the D peak after exfoliation (Figure S7e). However, we clearly observed that GR-related peaks disappeared through 2D/G in a specific region (Figure S7h). This can be attributed to the diversely distributed layers of our EG.

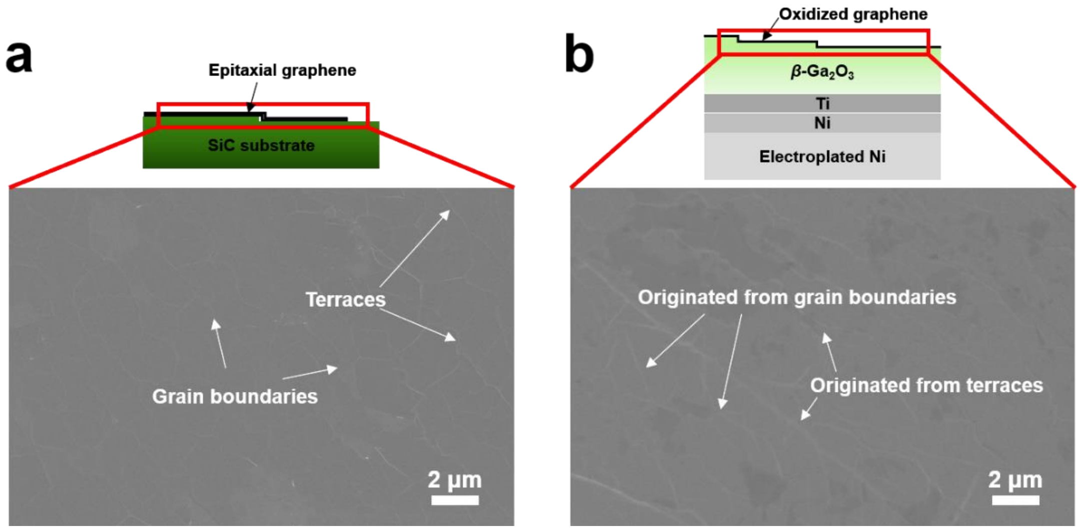

Figure S8. (a) Surface SEM images of the EG on SiC, and (b) back side of the β-Ga_2O_3 nanomembrane after exfoliation. Based on a comparison of SEM images after exfoliation, the surface morphology of the back side of the β-Ga_2O_3 was similar to the surface of the EG on SiC.

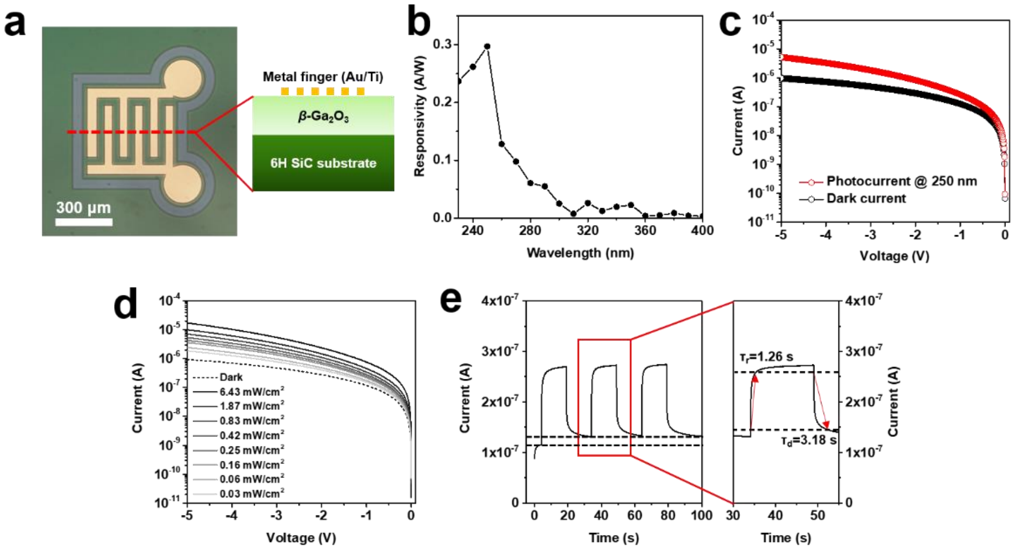

Figure S9. Lateral solar-blind photodetectors developed by using *β*-Ga_2O_3 layer grown on SiC. (a) Digital camera image of a lateral solar-blind photodetector (PD) made using two metal fingers on the same surface based on *β*-Ga_2O_3 grown on a bulk SiC substrate. The inset shows a cross-sectional schematic illustration of the lateral PD. (b) Responsivity of the lateral solar-blind PDs according to wavelength from 400 nm to 230 nm. (c) Current–voltage characteristics of the lateral solar-blind PDs under illumination @ 250 nm (0.25 mW/cm^2) (d) Current–voltage characteristics of the lateral solar-blind PDs depending on the power density of @ 250 nm illumination. (e) Time-dependent photoresponse of the lateral solar-blind PDs @ 250 nm illumination.

References

1. Ni, Z. H.; Chen, W.; Fan, X. F.; Kuo, J. L.; Yu, T.; Wee, A. T. S.; Shen, Z. X. Raman Spectroscopy of Epitaxial Graphene on a SiC Substrate. *Phys. Rev. B* **2008**, *77*, 115416.
